# Lower Gastrointestinal Bleeding, Vomiting, and Weight Stagnation in a Well-Appearing Neonate with Neonatal Transient Eosinophilic Gastroenterocolitis

**DOI:** 10.1055/a-2531-4618

**Published:** 2025-02-20

**Authors:** Bernat Servitje-Verdaguer, Clara Comalrena-de-Sobregrau-Martínez, Carmen-María Sánchez-Molina, Joan-Carles Ferreres-Piñas, Núria Torre-Monmany, Diana García-Tirado

**Affiliations:** 1 Department of Neonatology, Parc Taulí University Hospital, Sabadell, Barcelona; 2 Parc Taulí Research and Innovation Institute (I3PT-CERCA), Sabadell, Barcelona; 3 Department of Pediatric Gastroenterology, Hepatology, and Nutrition, Parc Taulí University Hospital, Sabadell, Barcelona; 4 Department of Pathology, Parc Taulí University Hospital, Sabadell, Barcelona

**Keywords:** hematochezia, eosinophilia, eosinophilic gastrointestinal disorders, neonatal transient eosinophilic colitis, food protein-induced allergic proctocolitis

## Abstract

This study presents a novel case of neonatal transient eosinophilic gastroenterocolitis, a proposed new entity causing upper and lower digestive symptoms and extensive gastrointestinal eosinophilic infiltration in newborns without a proven active food allergy. The condition's proposed pathophysiology and relationship to similar conditions, alongside clinical and therapeutic approaches, are also discussed.

Neonatal lower gastrointestinal bleeding must be thoroughly evaluated, as it may indicate a severe underlying disease. 1 Conditions such as necrotizing enterocolitis or intussusception often present in ill-appearing newborns, while differential diagnosis in well-appearing neonates includes anal fissures, swallowed maternal blood, coagulation disorders, and gastrointestinal food allergies (GIFAs). 1 2 3

GIFAs were once considered a common condition in neonates, but recent data with strict diagnostic criteria suggest that they are much rarer than previously thought, 4 5 down to <1%. 4 Non-IgE-mediated GIFAs are the most common type, 6 which includes food protein-induced enterocolitis syndrome (FPIES), food protein-induced allergic proctocolitis (FPIAP), and food protein-induced enteropathy (FPE). 6 7 8 The pathophysiology is not yet well-established but is considered multifactorial, involving an interplay between food allergens, genetic predisposition, and environmental factors. 6 9 Histological examination shows eosinophilic infiltration of the lamina propria of the affected digestive segment. 6 10 11 Most cases achieve clinical remission with an allergen-free diet and develop allergen tolerance by age 3. 6 12

Neonatal transient eosinophilic colitis (NTEC) recently emerged as an entity causing hematochezia in well-appearing newborns, 2 3 with clinical and histological similarities with FPIAP, 5 10 11 but lacking a proven allergic background, with negative oral challenge tests after symptom remission. 3 Few cases of NTEC have been reported so far, and these are self-limited, requiring no treatment. 5 12 No other neonatal gastrointestinal conditions resembling GIFAs but without a proven allergic component have been described.

In this study, we present the case of a well-appearing newborn who developed bloody diarrhea before any oral intake, with progression to vomiting and weight stagnation. Both FPIAP and NTEC were initially considered, but the patient was ultimately diagnosed as the first reported case of neonatal transient eosinophilic gastroenterocolitis (NTEGEC).

## Case Report

The patient was a Caucasian male newborn conceived naturally. The mother was a 32-year-old who was gravida 1, para 1, with a personal history of allergic asthma and had followed a vegan diet for 12 years. No other relevant family history was reported, and his older sibling had had no health issues. The pregnancy was well-controlled and uneventful. The patient was born at 35 weeks and 5 days via vacuum-assisted vaginal delivery. The amniotic fluid was meconium-stained. Venous umbilical pH was 7.01. Apgar scores were 8 at 1 minute and 9 at 5 minutes. His birth weight was 3,040 g.

**Fig. 1:**
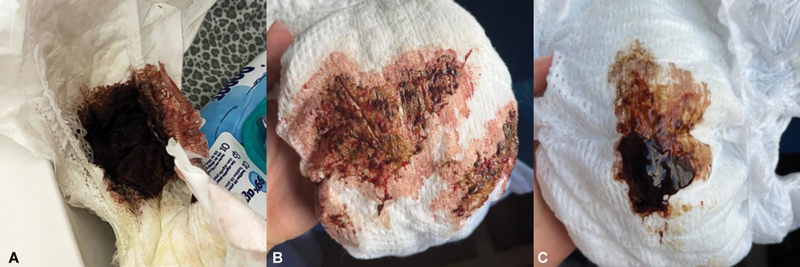
Features of the patient's stools during his first 24 hours of life: at 1 hour (A), 12 hours (B), and 16 hours (C).

In the first hour of life, prior to breastfeeding, the patient passed meconium with a melenic appearance, followed by copious bloody mucous diarrhea, totaling 20 stools within the first 48 hours ( Fig. 1 ). Despite this, the patient remained in good general condition, and his physical examination was unremarkable.

At 48 hours, despite the patient's good general condition and no signs of intestinal pneumatosis on abdominal X-ray, oral feeding was discontinued and replaced with parenteral nutrition due to concerns about possible necrotizing enterocolitis. Intravenous antibiotics (ampicillin and gentamicin) were administered for 72 hours until negative blood and urine cultures were obtained.

Blood analysis showed a hemoglobin level of 13.7 g/dL, leukocytosis of 24,490/μL with marked eosinophilia of 6,880/μL (28.1%), a normal platelet count of 305,000/μL, negative C-reactive protein, and procalcitonin, without other abnormalities. A stool PCR panel and culture ruled out infectious diarrhea. Abdominal ultrasonography was normal. Fecal alpha-1 antitrypsin was normal, while fecal elastase was decreased.

**Fig. 2:**
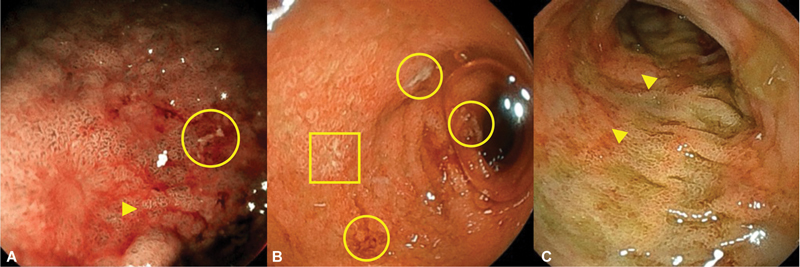
Macroscopic view of the mucosa of the stomach antrum (A, magnification with blue-laser imaging), second portion of the duodenum (B), and rectum (C) by endoscopy. Arrows, circles, and squares highlight some areas of erythematous mucosa, ulcers, and aphthae, respectively.

**Fig. 3:**
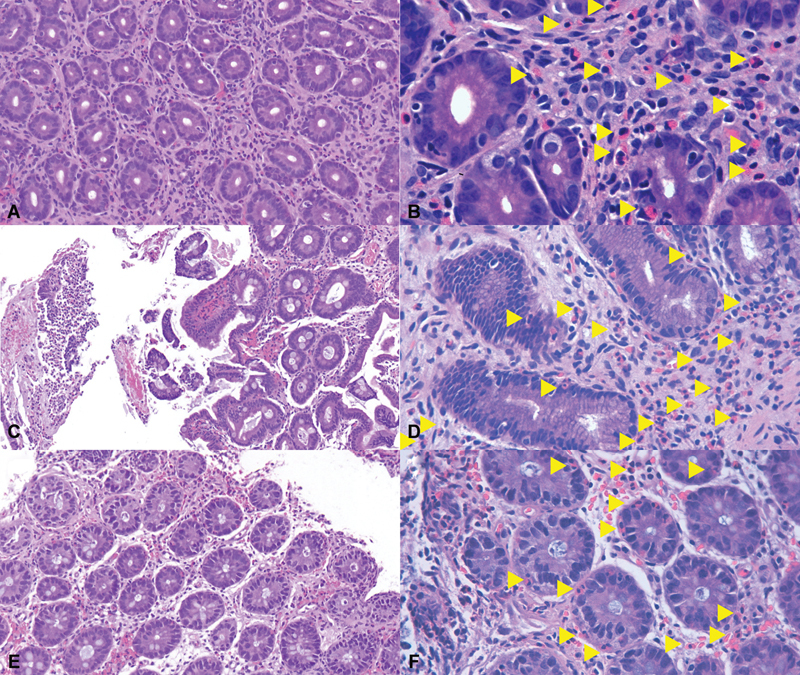
Histological view of biopsies from the stomach (A, B), duodenum (C, D), and colon (E, F). Arrows highlight some of the eosinophils.

Upper and lower digestive endoscopies were performed at 7 days. The esophagus appeared normal, whereas the stomach and duodenum were erythematous, friable, and ulcerated. The recto sigmoidoscopy showed diffuse erythema and erosions as well ( Fig. 2 ). Histologic examination revealed profuse eosinophilic infiltration of the lamina propria in the stomach (>200 eosinophils per high-power field), duodenum (118), and lower digestive tract (130), without any other identifiable changes ( Fig. 3 ).

Severe causes of hematochezia were ruled out. FPIAP and NTEC were the two main diagnoses initially considered, but histological findings were inconsistent, as both have exclusively colorectal involvement. The low stool elastase was attributed to transient exocrine pancreatic insufficiency due to malnourishment.

**Fig. 4.**
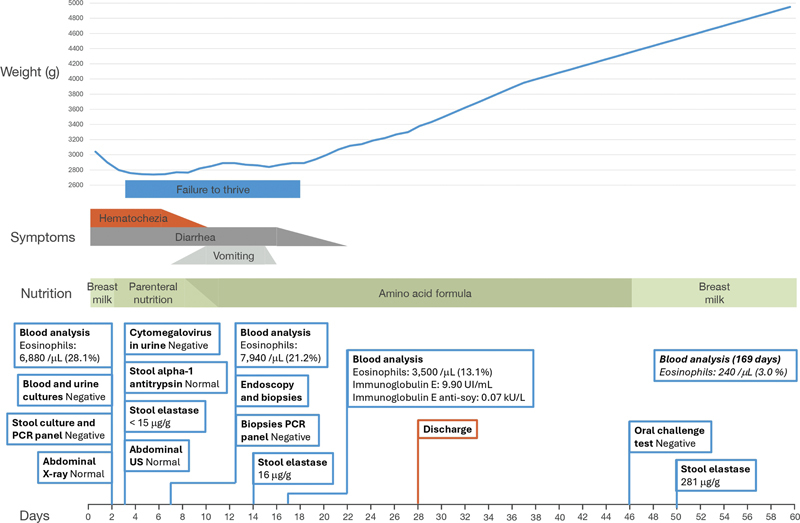
Summary of the patient's clinical course through his hospitalization, including weight, symptoms, nutrition, and complementary tests performed.

Enteral nutrition using an elemental amino acid formula was initiated at 8 days via a nasogastric tube. Caloric intake was gradually increased until parenteral nutrition was discontinued at 11 days. Stools stopped being bloody after 10 days. However, the patient then experienced daily vomiting, persistent diarrhea, and weight stagnation. Enteral nutrition was adjusted, and pancreatic enzyme replacement therapy was prescribed. At 16 days, vomiting ceased, and the patient's general condition gradually improved, although diarrhea persisted until 22 days. Blood analysis showed a steady decline in eosinophil count. Full oral feeding was achieved on day 26. The patient was discharged on day 28 with a normalized weight curve ( Fig. 4 ).

Outpatient follow-up was conducted. An oral challenge test with breast milk on day 46 was negative, ruling out an active GIFA. Breastfeeding without maternal dietary restrictions was indicated. Stool elastase normalized at 50 days, allowing pancreatic enzyme replacement therapy to be discontinued. The patient showed a favorable evolution, with a resolution of the eosinophilia (240/μL at 169 days), normal growth, and no recurrence of symptoms.

## Discussion and Review of the Literature

The present case was first thought to be NTEC. NTEC is a relatively novel term with limited data on its pathophysiology, as well as undefined diagnostic criteria and treatment protocols. Our patient exhibited strong clinical and histological similarities to previously reported cases, with alternative diagnoses having been excluded.

Table 1 summarizes all reported cases of diagnosed or suspected NTEC to date, 2 3 13 14 15 highlighting relevant history alongside clinical, analytic, and histological features compared to our case. Most cases involved moderate or late preterm newborns, which aligns with the current understanding of neonatal eosinophilic disorders, 16 17 as eosinophilia is more commonly observed in preterm newborns, reflecting hematopoiesis. 18 19 Amniotic fluid appears as “meconium-stained” in all cases, which is classically a sign of fetal distress but may also suggest in utero hematochezia and support the possibility of prenatal manifestations. 15 Prenatal abnormalities of the digestive system observed in some cases 13 14 15 may further suggest a prenatal onset.

**Table 1:** Comparative table of all reported cases of diagnosed or suspected NTEC, along with our case

	Ohtsuka et al 3		Tanaka et al 15	Debuf et al 2	Ota et al 14	Hoshi et al 13	This work
Relevant family history	None	None	Mother with seafood allergy Father with allergic asthma	None	Not stated	Not stated	Mother with allergic asthma
Maternal diet	Not stated	Not stated	Omnivorous	Not stated	Not stated	Omnivorous	Vegan
Prenatal ultrasound abnormalities	Not stated	Not stated	Dilated digestive tract High luminosity dots in the amniotic fluid	Not stated	Single bubble sign	Dilated stomach Dilated intestines Peri-intestinal strong echo patterns	None
Sex	Female	Male	Male	Male	Female	Female	Male
Ethnicity	East Asian	East Asian	East Asian	Black	East Asian	East Asian	White
Postmenstrual age at birth	34 wk and 4 d	37 wk and 1 d	36 wk and 1 d	36 wk and 5 d	36 wk and 2 d	31 wk and 2 d	35 wk and 5 d
Amniotic fluid	Not stated	Not stated	Meconium-stained	Meconium-stained	Meconium-stained	Meconium-stained	Meconium-stained
Onset of hematochezia	First few minutes	First 30 min	First minute	First few minutes	First few minutes	First minute	First hour
Upper digestive symptoms	Absent	Absent	Absent	Absent	Absent	Absent	Present
Failure to thrive	Absent	Absent	Absent	Absent	Absent	Absent	Present
Peak peripheral blood eosinophilia	9,014/μL	1,955/μL	10,080/μL	8,508/μL	3,800/μL	2,826/μL	7,940/μL
Type of digestive endoscopy performed	Lower	Lower	Not performed	Lower	Lower	Not performed	Upper and lower
Histological findings	Profuse eosinophilic infiltration in the descendent colon	Profuse eosinophilic infiltration in the descendent colon	Not performed	Profuse eosinophilic infiltration in the descendent colon	Profuse eosinophilic infiltration in the descendent colon	Not performed	Profuse eosinophilic infiltration in the stomach, the duodenum, and the descendent colon
Duration of hematochezia	8 d	8 d	6 d	First days of life	7 d	Not stated	10 d
Duration of parenteral nutrition	Not stated	Not stated	6 d	4 d	10 d	3 d	10 d
Age at discharge	Not stated	25 d	42 d	Not stated	Not stated	67 d	28 d
Oral challenge test with breast milk and normal maternal diet	Negative	Negative	Negative	Negative	Negative	Not performed	Negative

Abbreviations: d, day; NTEC, neonatal transient eosinophilic colitis wk, weeks.

Our patient shared most features with previously reported NTEC cases but raised new issues. In addition to a family history of asthma and allergy and the mother's strictly vegan diet, both potentially relevant to the condition, this case is also novel in that the patient is Caucasian, as only patients of Asian and African descent with NTEC have been reported. This is consistent with the current understanding that EGIDs are more prevalent in Asian populations but occur across all ethnicities. 7 10 However, our case displayed atypical clinical and histological features not previously described in NTEC, including vomiting and weight stagnation, along with eosinophilic infiltration extending from the stomach to the rectum rather than being limited to the colon. These discrepancies prompted a reassessment of the diagnosis, leading us to propose the term “NTEGEC” as a more accurate descriptor for the patient's condition.

We propose that NTEGEC represents a neonatal-onset EGID closely related to NTEC, both fitting within the spectrum of neonatal transient eosinophilic gastrointestinal disorders (NTEGIDs). 10 20 NTEGEC involves the upper and lower digestive tracts and may also have a prenatal onset, similarly to NTEC. While the incidence remains unknown, it is likely more common in late preterm and early term newborns, akin to NTEC. Symptoms may include early-onset bloody diarrhea, which may be followed by vomiting and weight stagnation, reflecting involvement of the entire gastrointestinal tract.

The pathophysiology of NTEGIDs, including NTEC and NTEGEC, is poorly understood. Some authors suggest that these conditions result from allergic reactions and predict a higher future incidence of atopy in affected patients, 20 21 although solid long-term data are lacking. This assumption is supported by recent findings highlighting significant clinical, genetic, and histological similarities between NTEC and FPIAP, 22 23 suggesting a shared pathophysiology potentially involving in utero food sensitization, 22 23 24 namely cow's milk, 25 even if this remains a controversial topic. According to this theory, NTEGEC would represent an early-onset, rapidly remitting allergic condition, even if an active GIFA may not be demonstrable due to rapid tolerance acquisition; for instance, 52% of patients with infantile-onset GIFAs achieve allergen tolerance by 1 year of age, 23 and NTEGEC patients are believed to develop tolerance even earlier.

Nomura et al 11 identified four clusters in non-IgE-mediated GIFAs based on symptoms. In this classification, NTEC aligns to cluster 4, as does FPIAP, 20 22 while NTEGEC fits into cluster 1, which includes FPIES. 11 22 Suzuki et al 20 further proposed that cluster 1 “might comprise a distinct patient group” and could histologically represent a “neonatal-onset EGID.” Accordingly, NTEC and NTEGEC might be neonatal variants of FPIAP and FPIES, 26 respectively.

However, there are alternative pathophysiological theories for NTEGIDs that do not involve allergic mechanisms. Other known causes of eosinophilia in well-appearing newborns include medications, perinatal distress, bronchopulmonary dysplasia, hypereosinophilic syndrome, anabolism, immunodeficiencies, adrenal insufficiency, catheters, hyperalimentation, and malignancies. 18 27 28 29 In our case, perinatal distress was present and is, therefore, an important factor to consider in the context of neonatal immune responses.

Neonatal immunology is characterized by certain particularities due to the immaturity of the immune system. 30 Immune responses in newborns tend to be weak, with a predominance of short-term Th2-mediated pathways, 30 31 32 which are associated with antibody-mediated immunity, eosinophilia, and atopy. 30 As such, almost any fetal or perinatal adverse event, including perinatal distress, can trigger a Th2-mediated eosinophilic response, which may occasionally be misinterpreted as an allergic phenomenon. From this perspective, NTEGEC might represent a transient immune reaction to perinatal stressors without any underlying allergic significance.

The management of NTEGIDs is hindered by the limited evidence available to back up clinical and therapeutic decisions. Still, there are key considerations that we believe merit emphasis. First, we consider that a comprehensive history and repeated thorough physical examinations are critical when addressing neonatal lower gastrointestinal bleeding, particularly to exclude severe conditions.

We propose that a diagnosis of NTEGID be established when eosinophilic infiltration of the gastrointestinal tract is demonstrated alongside compatible symptoms, provided other conditions have been ruled out and an oral challenge test to breast milk with a normal maternal diet yields negative results. Cases presenting solely with lower digestive symptoms and colonic eosinophilic infiltration are more consistent with NTEC, while cases involving both upper and lower gastrointestinal symptoms and more extensive gut involvement are indicative of NTEGEC. As evidence advances in this field, there could be a shift toward a less invasive diagnostic approach, potentially with more restrictive indications for endoscopy.

Currently, no standardized treatment protocols exist. Most cases have been managed with total parenteral nutrition followed by a gradual introduction of an enteral diet using an amino acid formula until an oral challenge test allowed the reintroduction of breast milk. After that, no further treatment is typically required, as NTEGIDs are transient conditions. However, we recommend outpatient follow-up to ensure appropriate evolution and development. With additional evidence in the future, a less invasive therapeutic approach may become feasible, favoring a wait-and-see strategy in mild, short cases.

## Conclusion

In conclusion, we describe NTEGEC as a neonatal disorder causing both upper and lower digestive symptoms, closely related to NTEC. Emerging data suggest that NTEGIDs, including NTEC and NTEGEC, may represent prenatal-onset, rapidly resolving non-IgE-mediated GIFAs. However, this remains controversial, as eosinophilic responses in newborns might be triggered by various adverse events unrelated to allergy. Many pathophysiological, clinical, and therapeutic aspects of this topic remain unexplored and warrant further research.
